# Variability of Metabolic Rate and Distribution Volume Quantification in Whole-Body Parametric PATLAK [^18^F]-FDG PET/CT—A Prospective Trial in Patients with Lung Cancer

**DOI:** 10.3390/diagnostics15131719

**Published:** 2025-07-05

**Authors:** Stephan Ursprung, Lars Zender, Patrick Ghibes, Florian Hagen, Konstantin Nikolaou, Christian la Fougère, Matthias Weissinger

**Affiliations:** 1 Department of Diagnostic and Interventional Radiology, University Hospital Tuebingen, 72076 Tuebingen, Germanymatthias.weissinger@med.uni-tuebingen.de (M.W.); 2 Department of Medical Oncology and Pneumology (Internal Medicine VIII), University Hospital Tuebingen, 72076 Tuebingen, Germany; 3 iFIT-Cluster of Excellence, Eberhard Karls University Tuebingen, 72076 Tuebingen, Germany; 4 German Cancer Consortium (DKTK), Partner Site Tuebingen, 72076 Tuebingen, Germany; 5 Department of Nuclear Medicine and Clinical Molecular Imaging, University Hospital Tuebingen, 72076 Tuebingen, Germany; 6 Department of Nuclear Medicine, Marienhospital Stuttgart, 70072 Stuttgart, Germany

**Keywords:** whole-body, dynamic PET, parametric FDG, Patlak, FDG, PET/CT

## Abstract

**Background**: The recent introduction of whole-body positron emission tomography/ computed tomography (PET/CT) scanners and multi-bed, multi-time point acquisition technique enable calculating fluorodeoxyglucose (FDG) kinetics in the whole body. However, validating parametric, Patlak-derived data is difficult on phantoms. **Methods**: This prospective study investigated the effect of quantification methods mean, max, and peak on the metabolic rate (MR-FDG) and distribution volume (DV-FDG) quantification, as well as the diagnostic accuracy of parametric Patlak FDG-PET scans in diagnosing lung lesions and lymph node metastases, using histopathology and follow-up as reference standards. Dynamic whole-body FDG PET was acquired for 80 minutes in 34 patients with indeterminate lung lesions and kinetic parameters extracted from lung lesions and representative mediastinal and hilar lymph nodes. **Results**: All quantification methods—mean, max, and peak—demonstrated high diagnostic accuracy (AUC: MR-FDG: 0.987–0.991 and 0.893–0.905; DV-FDG: 0.948–0.975 and 0.812–0.825) for differentiating benign from malignant lymph nodes and lung lesions. Differences in the magnitude of MR-FDG (−4.76–14.09) and DV-FDG (−10.64–46.10%) were substantial across methods. Variability was more pronounced in lymph nodes (MR-FDG: 1.37–3.48) than in lung lesions (MR-FDG: 3.31–5.04). The variability was lowest between mean and max quantification, with percentage differences of 40.87 ± 5.69% for MR-FDG and 39.26 ± 7.68% for DV-FDG. **Conclusions**: The choice of method to measure MR-FDG and DV-FDG greatly influences the results, especially in smaller lesions with large and systematic differences. For lung lesions, a conversion factor between mean and max methods of 40% provides acceptable agreement, facilitating retrospective comparisons of measurements, e.g., in meta-analyses.

## 1. Introduction

Positron emission tomography (PET) using the glucose analog [^18^F]Fluorodeoxyglu- cose ([^18^F]FDG) is the gold standard for staging, assessing treatment response, and detecting recurrence in many solid tumors [1]. Glucose metabolism is significantly increased in most fast-growing malignant tissues, enabling non-invasive differentiation between benign and malignant disease. PET may quantify the absolute activity concentration of [^18^F]FDG in tissue at defined time points, typically 60–90 minutes post injection [1]. However, absolute quantification requires cross-calibration of image counts reconstructed from raw data with known activity concentrations. Therefore, simplified semi-quantitative Standardized Uptake Values (SUVs) are commonly utilized in clinical oncology [1,2].

SUVs can be determined without cross-calibration and normalize the measured tumor activity to the injected tracer activity and the patient’s body weight [2,3]. Empirically defined SUV thresholds help physicians to distinguish between malignant and benign tissues for many tumor types [4,5,6,7].

Beyond single time-point [^18^F]FDG concentrations, tracer kinetics between different tissue compartments can be derived from kinetic modeling of continuous PET data using the Patlak method [8,9]. The approach uses a two-compartment model of glucose metabolism, quantifying the [^18^F]FDG influx rate into the tissue (metabolic rate = MR) and the distribution volume (DV) of FDG in the tissue. These parameters provide additional information beyond SUV [8,9].

Calculating SUV, MR, and DV is complex and affected by hardware variations, random coincidences, detector dead time, photon attenuation, and scatter [10]. When confounding factors are properly addressed, PET yields a highly consistent quantification with variations around 10% [11,12].

For clinicians, [^18^F]FDG quantification and the application of cutoff values can facilitate objective image evaluation [4,13]. However, in oncological diagnostics and clinical studies, the choice of a tumor delineation method and activity threshold is crucial to track tumor uptake during therapy. For example, a 30% reduction in SUV is considered clinically significant and informs the decision on tumor response according to the PERCIST (Positron Emission Tomography Response Criteria in Solid Tumors) criteria [14]. Moreover, correctly applied empirical SUV and MR cutoff values differentiate neoplastic and inflammatory diseases as well as experts [4,6,13,15,16].

The choice of SUV quantification method, whether SUV_max_ (voxel with the highest activity in a VOI), SUV_mean_ (average value in a defined VOI and threshold relative to the voxel with the highest activity), or SUV_peak_ (average value within a 1 mL sphere centered in the highest activity within the VOI), significantly affects the SUV’s magnitude and cutoff value [10,17]. Variations among these methods can be misinterpreted as changes in tumor glucose metabolism or can lead to an under- or overestimation of the metabolic tumor volume when using threshold-based auto-segmentation.

Therefore, this study investigated the effect of quantification methods mean, max, and peak on the metabolic rate and distribution volume quantification as well as the diagnostic accuracy of parametric Patlak FDG-PET scans in diagnosing lung lesions and lymph node metastases.

## 2. Materials and Methods

### 2.1. Study Design

**Figure 1:**
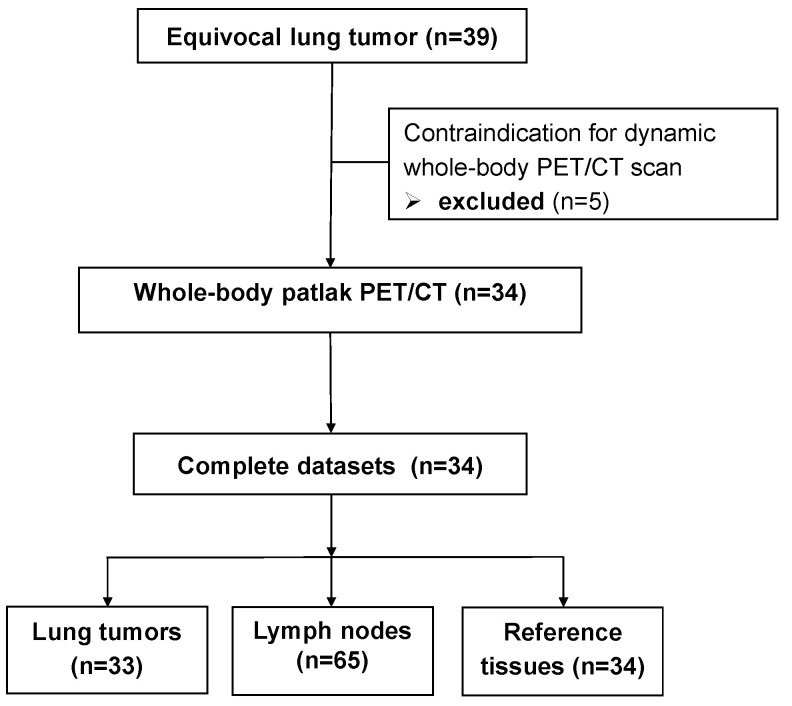
Consolidated Standards of Reporting Trials (CONSORT) flow diagram for patient enrollment.

This prospective single-center trial recruited patients with newly diagnosed indeterminate pulmonary lesions with a clinical indication for [^18^F]FDG-PET/CT. Eligible patients were at least 18 years of age, had capacity to give consent, and achieved adequate blood glucose control as detailed below. Iodinated contrast was administered only if the renal function was sufficient (eGFR ≥ 30 mL/min/1.73 m^2^). Only patients scheduled as the first examination in the morning were eligible to allow for sufficient time for dynamic imaging. Exclusion criteria were known etiology of the lung disease, prior disease-specific treatment, inability to remain motionless during imaging, claustrophobia, pregnancy and breast-feeding. Patients requiring medication to manage pain or claustrophobia were not considered for this study. Between June 2019 and April 2022, thirty-three consecutive patients were enrolled (Figure 1; Consolidated Standards of Reporting Trials (CONSORT) flow diagram). The clinical trial was approved by the Institutional Review Board (registry No. 333/2019BO2) and is listed in the German Clinical Trial Register (DRKS-ID: DRKS00017717). The study was carried out in accordance with the relevant guidelines and regulations. All patients provided written informed consent.

### 2.2. PET/CT Examination Protocol

Patients followed a zero-sugar and carbohydrate fasting protocol for at least 6 h before imaging. They were instructed to drink only clear fluids and consume no food. Body weight, height, and blood glucose levels were measured prior to tracer injection. Blood glucose levels needed to be below 140 mg/dL (7.8 mmol/L), and no insulin was to be administered in the six hours preceding the procedure. [^18^F]FDG was adjusted for body weight (4.0 ± 0.6 MBq/kg) [18]. All patients were scanned on the same integrated PET/CT device (Biograph mCT Flow 128, Software Version VG70A, Siemens Healthineers, Erlangen, Germany). The CT was based on the Definition AS platform, using a 128-slice energy-integrating detector. The PET detector had an axial field of view of 216 mm and used Lutetium Oxyorthosilicate (LSO) crystals of 4×4×20 mm. Patients were positioned supine on a vacuum mattress with their arms raised to reduce motion artifacts and were instructed to breathe steadily and shallowly during the procedure. A diagnostic CT scan with adjustable tube voltage (CARE kV 120–140 kV) and current (CARE Dose 4D 40–280 mAs) was performed just before PET. Unless contraindicated, iodinated contrast agent (80–100 mL Ultravist 370, Bayer, Leverkusen, Germany dosed to body weight) was administered at 2.5 mL/s followed by a 25 mL saline chaser. The acquisition commenced 70 s after beginning the contrast injection to capture a portal-venous phase CT from vertex to mid-thigh.

Dynamic PET acquisition began simultaneously with intravenous tracer injection and lasted 80 min. Initial imaging of the cardiac region captured the arterial input function for approximately 6 min. Subsequently, dynamic whole-body imaging from the skull to mid-thigh was performed for approximately 74 min using a continuous scanning technique (FlowMotion, Siemens Healthineers) in 3D list mode with 17 scanner passes, as described in detail by Karakatsanis et al. and Rahmim et al. [19,20,21].

Image data were reconstructed into 43 time frames (12 × 5 s, 6 × 10 s, 8 × 30 s, 7 × 180 s, and 10 × 300 s) with increasing frame durations. Time-activity curves (TACs) were derived from cylindrical regions of interest automatically generated in the descending aorta using ALPHA (automated learning and parsing of human anatomy) as implemented in the vendor’s software (VG70A, Siemens Healthcare GmbH, Erlangen, Germany).

### 2.3. Reconstruction and Postprocessing

Dynamic PET data, including cardiac and whole-body (WB) regions, were reconstructed using OSEM 3D (ordered subset expectation maximization) reconstruction with point-spread-function (PSF) and time-of-flight (TOF). Reconstruction was performed with 2 iterations with 21 subsets, a 200 × 200 matrix, and a 5 mm Gaussian filter. Passes 12–17 of the WB data and the corresponding TACs were used for Patlak reconstructions, applying identical reconstruction parameters with vendor-specific software (VG70A, Siemens Healthcare GmbH). Static whole-body images, as part of the standard of care, were reconstructed from passes 15–17 of the WB data using ultraHD-PET (PSF + TOF). These images were reconstructed with two iterations, 21 subsets, a 400 × 400 matrix, and a 2 mm Gaussian filter.

[^18^F]FDG kinetics were analyzed using a two-compartment model with linear Patlak analysis, generating whole-body parametric images of Patlak slope and intercept [8,9], as described in detail by A. M. Smith et al. [22]. The Patlak slope, representing the constant influx rate of [^18^F]FDG (Ki_mean_; mL/min/100 mL = 0.01 × min^−1^), was multiplied by blood glucose levels to calculate the metabolic rate of [^18^F]FDG (MR-FDG_mean_), given in µmol/(min × 100 mL). The Patlak intercept, given as a percentage, represents the distribution volume of free [^18^F]FDG (DV-FDG_mean_) in reversible compartments and fractional blood volume [13]. Semi-quantitative measurements were performed in static images using SUV_max_, SUV_mean_ (50% isocontour), and SUV_peak_ (1 mL sphere).

### 2.4. Image Evaluation and Segmentation

Parametric images were reconstructed using dedicated software (syngo.via^®^ 8.2, Siemens Healthineers). Volumes of interest (VOIs) were manually placed in lung lesions and representative mediastinal and hilar lymph nodes on fused PET/CT images and verified by a board-certified expert in nuclear medicine and radiology with over five years of experience in PET/CT imaging. VOIs were propagated to the Ki dataset, DV-FDG and the static PET images for quantification. Tumor-free liver parenchyma was defined as reference tissue. For liver parenchyma quantification, a 100 mL VOI was placed in tumor-free liver tissue in the right lobe, avoiding large portal vessels. If necessary, manual co-registration was performed to ensure adequate alignment.

### 2.5. Reference Standard

Final diagnoses were established through histological analysis, extended follow-up, or a consensus decision by the institutional tumor board. Only thoracic lymph nodes with histopathological confirmation, as well as those identified as enlarged on CT and/or PET-positive with follow-up confirmation, were included in the statistical analysis.

### 2.6. Statistical Analysis

Mean differences between groups, features, or methods were evaluated using a two-sided Student’s *t*-test. Levene’s test was performed to assess the equality of variance before conducting *t*-tests. One-way ANOVA was used to compare histological classifications (inflammatory/benign or malignant) for MR and DV.

Agreement between different DV-FDG and MR-FDG quantification methods was evaluated using Bland–Altman plots [23]. Mean values were plotted on the X-axis, while absolute and percentage differences were shown on the Y-axis. The limits of agreement were defined as the mean difference ±1.96 standard deviations. Post hoc comparisons were adjusted using Tukey’s honest significant difference method. Results were deemed significant at *p* < 0.05.

Optimal cutoff values were determined by minimizing the sum of false-negative and false-positive rates. The diagnostic accuracy was measured using the area under the receiver operating curve (AUC). AUCs were compared using DeLong’s test. Statistical analyses were conducted using SPSS Statistics 28.0 (IBM Inc., Armonk, NY, USA), MATLAB R2022b (The MathWorks, Inc., Natick, MA, USA), and MS Excel 2019 (Microsoft Corporation, Redmond, WA, USA).

## 3. Results

### 3.1. Patient Cohort

The prospective study recruited from 2015 to 2022, and 34 consecutive patients were enrolled (14 female, 20 male). The mean age was 64.5 ± 10.1 years. Mean BMI was 26.2 ± 5.3 with a mean height of 171.7 ± 12.4 cm and mean weight of 77.7 ± 19.1 kg. Glucose levels were 5.5 ± 0.95 mmol/L at the time of [^18^F]FDG injection with no significant difference between males and females. No patient had a history of diabetes mellitus.

The injected FDG activity was 4.0 ± 0.7 MBq/kg body weight, resulting in a total activity of 194–424 MBq.

### 3.2. Lesion Characteristics

Overall, 65 lymph nodes (2.0 ± 6.4 mL), 33 lung lesions (32.5 ± 58.0 mL) and liver parenchyma (100 ± 0.0 mL) were analyzed. Table 1 and Table 2 summarize mean SUV, MR-FDG and DV-FDG of the lymph nodes and lung lesions, respectively.

### 3.3. Comparison of Quantification Methods: Metabolic Rate

**Figure 2:**
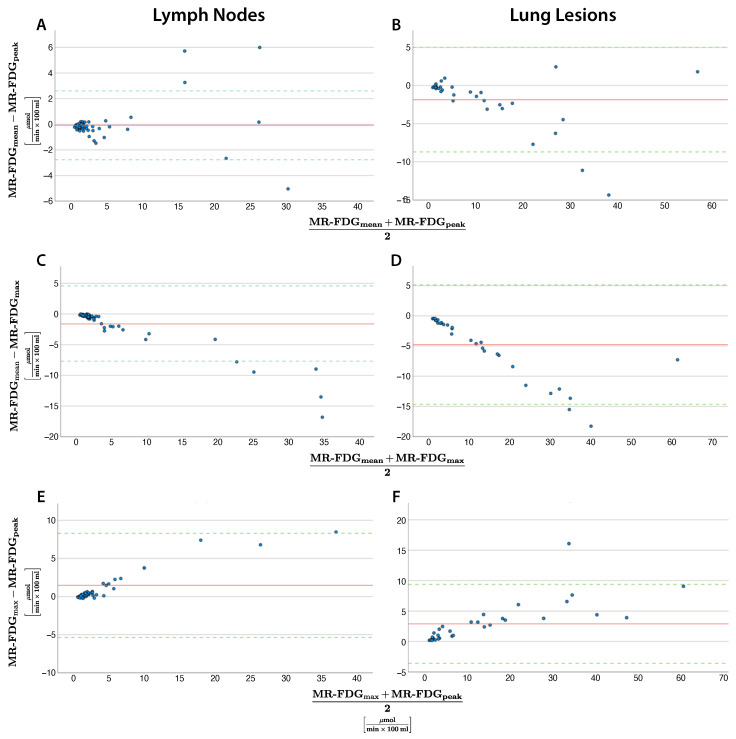
Bland–Altman plots comparing quantification methods mean, max and peak for metabolic rate (MR) in lymph nodes (**A**,**C**,**E**) and lung lesions (**B**,**D**,**F**). The red line indicates the mean difference and the green line 1.96 standard deviations.

Mean vs. Peak: At low MR-FDG (<10 µmol/(min × 100 mL)), measurements agree well, without any systematic bias, for lymph nodes and lung lesions. However, MR-FDG_peak_ is systematically higher in pulmonary lesions (mean difference −1.85 ± 3.49 SD), exceeding the lower limit of agreement of −8.70 µmol/(min × 100 mL) at a metabolic rate >30 µmol/(min × 100 mL). Variability was high in lymph nodes without systematic bias (mean difference: −0.07 ± 1.37 SD) (Figure 2A,B).

Max vs. Mean: The MR-FDG_max_ was systematically higher in lung lesions (mean difference +4.76 µmol/(min × 100 mL)) and lymph nodes (+1.55 µmol/(min × 100 mL)).

**Figure 3:**
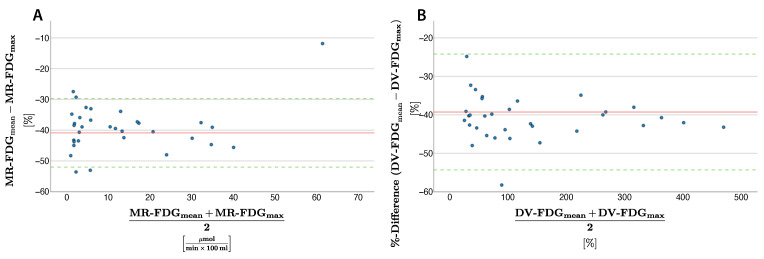
Bland-Altman plots comparing the percentage difference in metabolic rates (**A**) and distribution volume (**B**) between mean and max in lung lesions. The red line indicates the mean difference and the green line 1.96 standard deviations.

The range of agreement (−14.65—5.13) was exceeded at a metabolic rate >30 µmol/ (min × 100 mL) for lung lesions (Figure 2C,D). MR-FDG_max_ was on average 40.87% ± 5.69% higher, with decreasing relative differences as absolute values increase (Figure 3A).

Max vs. Peak: MR-FDG_max_ was on average 1.47 µmol/(min × 100 mL) higher in lymph nodes and 2.91 µmol/(min × 100 mL) higher in lung lesions (relative difference: (29.10 ± 12.20%). Differences between the methods increased in proportion with increasing MR, but remained within the range of agreement for lymph nodes (−5.21–8.15 µmol/(min × 100 mL)) and for all but one outlier lung lesion (−3.58–9.39 µmol/(min × 100 mL)) (Figure 2E,F).

The data are detailed for lymph nodes and lung lesions in Table 3 and Table 4, respectively.

### 3.4. Comparison of Quantification Methods: Distribution Volume

**Figure 4:**
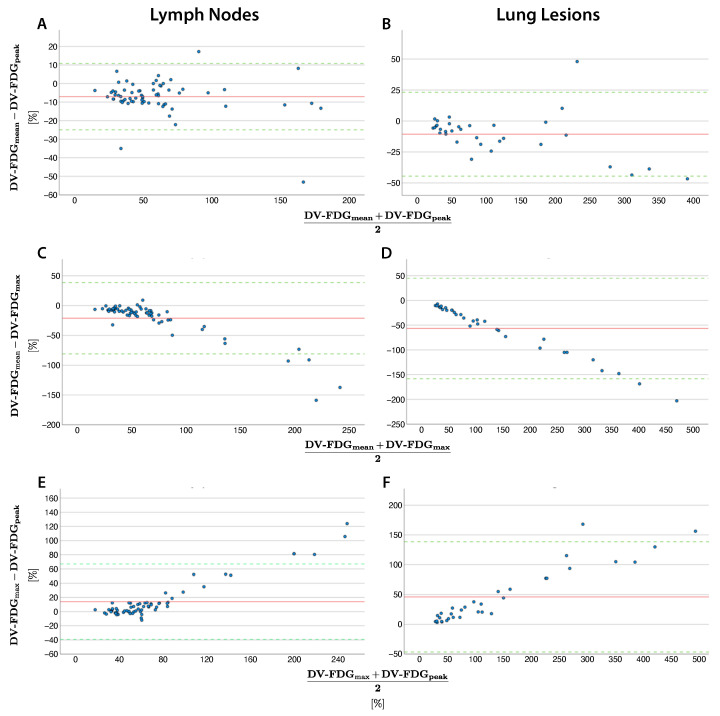
Bland–Altman plots comparing quantification methods mean, max and peak for distribution volume (DV) in lymph nodes (**A**,**C**,**E**) and lung lesions (**B**,**D**,**F**). The red line indicates the mean difference and the green line 1.96 standard deviations.

Mean vs. Peak: DV-FDG_peak_ was higher in lymph nodes (mean difference 7.06% ± 9.13%) and lung lesions (10.64% ± 17.24%). The dispersion around the mean difference was broad for lymph nodes (−24.95%–10.82%) and lung lesions (−44.44%–23.15%), without a clear trend (Figure 4A,B; Table 3 and Table 4).

Max vs. Mean: DV-FDG_max_ was significantly higher in lymph nodes (mean difference 21.16% ± 30.38%) and lung lesions (56.74% ± 51.83%). Differences increased linearly with increasing DV-FDG. Percentage transformation achieved the lowest SD of 7.68% in lung lesion measurement with a mean difference of −39.26% (Figure 3A). However, in lymph nodes, the SD was significantly higher (17.45%) and the agreement range was exceeded (−80.71%–38.39%) beginning at DV-FDG of 200%. For lung lesions, the agreement range was exceeded at around 300%, with an overall higher agreement range in lung lesions (−54.31%–24.21%) (Figure 4C,D).

Max vs. Peak: DV-FDG_max_ was higher in lymph nodes (mean difference 14.09% ± 27.13%) and lung lesions (46.10% ± 47.21%). The difference between the two quantification methods increased proportionally with the rising distribution volume, resulting in average relative differences of 15.66 ± 17.70% in lymph nodes and 25.29 ± 14.58% in lung lesions (Figure 3). The agreement range was exceeded above ∼200% DV-FDG for lymph nodes and ∼300% for pulmonary nodules, with a broad range of agreement for lymph nodes (−39.10%–67.28%) and pulmonary nodules (−46.43%–138.62%; Figure 4E,F).

The data are summarized for lymph nodes and lung lesions in Table 3 and Table 4, respectively.

### 3.5. Comparison of Quantification Methods: Liver Parenchyma

**Figure A1:**
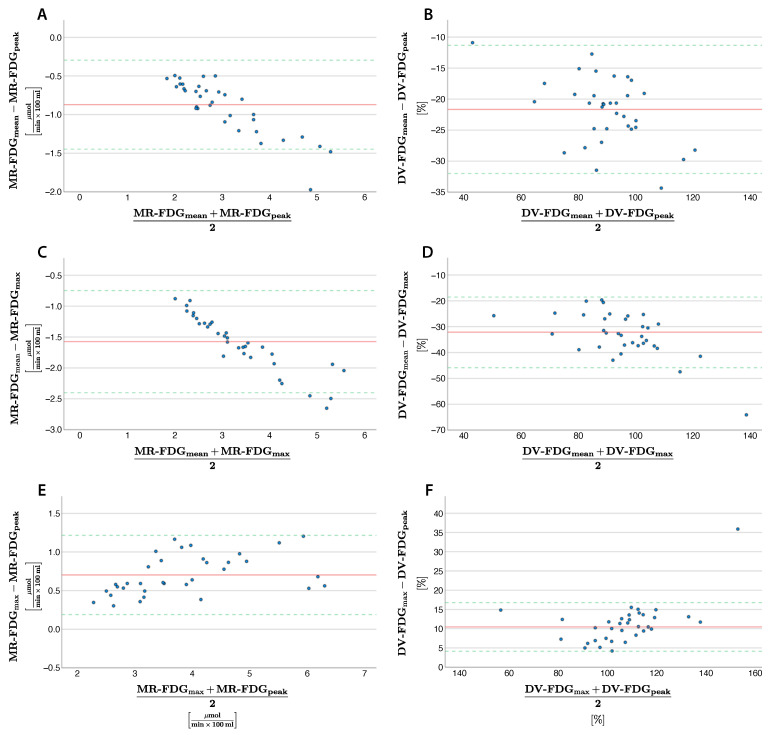
Bland–Altman plots comparing quantification methods mean, max and peak for metabolic rate (MR; **A**,**C**,**E**) and distribution volume (DV; **B**,**D**,**F**) in reference liver tissue. The red line indicates the mean difference and the green line 1.96 standard deviations.

Considering the liver parenchyma as a representative for large volumes of 100 mL, peak and max quantified 0.87 ± 0.29 µmol/(min × 100 mL) or 1.57 ± 0.42 µmol/(min × 100 mL) higher metabolic rates compared to mean, respectively, with a nearly linear trend line of increasing differences in case of increasing metabolic rates. Directly comparing max and “peak,” max showed a significantly higher metabolic rate than peak (mean: 10.46 ± 3.22, *p* < 0.01) with a slight trend of an increasing difference at higher metabolic rates (Figure A1 in Appendix A).

Quantification of hepatic DV-FDG using the peak and max methods resulted in 21.66% ± 5.27% and 32.11% ± 6.93% higher DV than the mean method. Specifically, the max method resulted in 10.45% ± 3.22% higher values compared to the peak method. Overall, Bland–Altman plots depict a diffuse fluctuation around the mean values without a clear trend of increasing deviation with increasing distribution volumes.

### 3.6. Diagnostic Accuracy and Cutoff Value: Lymph Nodes

The area under the receiver operating characteristic curve (AUC), a measure of diagnostic accuracy, showed very high accuracy and no significant differences among the three quantification methods for both MR-FDG and DV-FDG measurements (Table 5).

However, at a clinically optimal sensitivity defined at 92.0%, the cutoff values for different quantification methods differ significantly. For MR, the mean method has a optimal cutoff value at 3.4 µmol/(min × 100 mL) with a specificity of 98.1%, max cutoff (5.6 µmol/(min × 100 mL); specificity: 98.1%) and peak cutoff (4.0 µmol/(min × 100 mL); specificity: 96.2%).

The DV-FDG_mean_ has an optimal cutoff value at 60.3% (specificity: 81.1%), DV-FDG_max_ has a optimal cutoff at 82.4% (specificity: 91.6%) and DV-FDG_peak_ at 70.5% (specificity: 90.6%).

### 3.7. Diagnostic Accuracy and Cutoff Value: Lung Lesions

**Figure 5:**
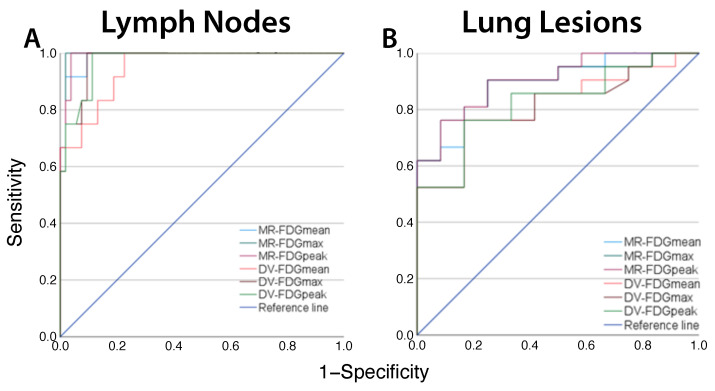
Receiver operating characteristic (ROC) curve for the prediction of malignancy in lymph nodes (**A**) and lung lesions (**B**) based on different quantification methods of the metabolic rate (MR) and distribution volume (DV) of [^18^F]FDG).

The AUC analysis revealed no significant differences in the diagnostic accuracy between the quantification methods for MR-FDG and DV-FDG (Figure 5, Table 6).

At a pre-defined sensitivity of 90.5%, all quantification methods revealed comparable specificity (75.0%). However, cutoff values were significantly different for mean, max and peak (2.3, 3.5 and 2.5 µmol/(min × 100 mL)). At a pre-determined specificity of 90.5%, MR-FDG cutoff values differed significantly: mean method: 2.3 µmol/(min × 100 mL) (specificity: 75.0%), max: 3.5 µmol/(min × 100 mL) (specificity: 75.0%), and peak: 2.5 µmol/(min × 100 mL) (specificity: 75.0%). The optimal DV-FDG cutoff was found for the mean at 29.6% (specificity: 41.7%), for max at 41.4% (specificity: 25.0%) and for peak at 33.4% (specificity: 33.3%).

## 4. Discussion

Dynamic total-body imaging using Patlak PET enables quantification of glucose kinetics across tissue compartments simultaneously for all organs. This study confirms a high diagnostic accuracy of MR and DV of FDG in differentiating benign and malignant lung lesions and lymph nodes with histology and follow-up as reference standards. However, technical and statistical variations affect dynamic parameter quantification that cannot currently be assessed in phantoms, thus requiring in vivo evaluation. This study was the first to systematically analyze variations in Patlak PET quantification, revealing a significant impact on MR-FDG and DV-FDG, and thus sensitivity and specificity when applying cut-off values.

Recent advances in PET technology—including multi-bed, multi-time point acquisition, flow-motion technique, and long-axis field of view scanners—facilitate whole-body FDG kinetics quantification and its integration into routine clinical workflows [23].

Our results align with findings in the literature and our previous work, confirming the potential diagnostic utility of MR-FDG and DV-FDG in differentiating between malignant and benign findings [16,24]. This diagnostic value has now also been validated for the different quantification methods (mean, max, and peak).

Sensitivity and specificity in our study are comparable to those reported in the limited literature for lung carcinomas and lymph node metastases [24]. Specifically, Inoue et al. highlighted the utility of MR-FDG in distinguishing malignant lung lesions from pulmonary sarcoidosis [24], reporting lower MR-FDG in sarcoidosis and improved diagnostic accuracy compared to SUV [24]. The MR-FDG of malignant lesions (mean 3.84 ± 1.70 µmol/(min × 100 mL)) fell within two standard deviations of our results (14.31 ± 10.13 µmol/(min × 100 mL)) [24,25,26,27]. Numerical differences in MR-FDG may reflect differences in the patient cohorts. Inoue’s study focused on metastases from gastrointestinal tract cancers and breast cancer, which exhibit lower glucose metabolism and FDG uptake than primary lung lesions [28]. Lung carcinomas accounted for only ∼6% of their cohort. A breakdown of MR-FDG by tumor type is not available.

The optimal MR-FDG cutoff for lung lesions was 3.4 µmol/(min × 100 mL) in this study, slightly higher than the cutoff of 2.855 µmol/(min × 100 mL) reported in prior research [24].

Beyond diagnosis, Patlak PET analysis has the potential to predict therapeutic response. For example, Wang et al. demonstrated that Patlak Ki (influx rate) correlated positively with the response to immuno-chemotherapy in locally advanced non-small cell lung cancer [29]. However, their study reported only Ki, precluding direct comparison.

As dynamic PET parameters like MR-FDG and DV-FDG are new to clinical imaging, experience and validation are limited at many PET/CT sites. Therefore, validated cutoff values can facilitate image interpretation. Standardization is critical to minimize the effect of biological, technical, and statistical factors and ensure reproducible measurements. The value of standardization in FDG-PET is well established for SUV measurements, where effects of blood glucose levels, fasting time, and uptake intervals are controlled [10]. Furthermore, technical and statistical influences on SUV measurements are well-documented in phantom studies. However, the time-dependent tissue kinetics of [^18^F]FDG is challenging to simulate in a model, thus necessitating in vivo analysis.

In this study, we defined cutoff values for detecting lymph node metastases and malignant lung lesions using dynamic Patlak PET and validated them against histology and follow-up. The cutoff values varied significantly depending on the quantification method, and switching quantification methods without adjusting cutoffs led to significant changes in sensitivity and specificity. The variability among quantification methods approached the thresholds defining response or progression in PERCIST and is thus highly clinically relevant.

Our data indicate that variability in Patlak PET quantification depends on the lesion size and the magnitude of MR-FDG and DV-FDG, similar to findings for SUV [10]. For lymph nodes, representing small volumes, MR-FDG and DV-FDG showed significant variability between all quantification methods, with standard deviations ranging from 14% to 18%. Despite this variability, diagnostic accuracy in differentiating benign lymph nodes from metastases was high, with specificities of 96.2–98.1%. Correction factors for small objects appear insufficiently precise, highlighting the need for method-specific cutoff values.

For lung lesions, the two most established quantification methods, mean and max, exhibit systematic deviations but minimal percentage fluctuations. On average, the max method quantifies MR-FDG and DV-FDG values approximately 40% higher than the mean method, with an acceptable standard deviation of 5.7 µmol/(min × 100 mL) or 7.6%. This conversion may be used as a correction factor, which might be particularly valuable in meta-analyses. Nevertheless, for clinical reading, individualized cutoff values tailored to the quantification method should be applied.

### Limitations

The study’s dynamic imaging acquisition lasted 60 min post-tracer injection. For high-throughput clinical settings, this duration is feasible only for initial patients during uptake periods of subsequent cases. Shortened protocols have been proposed but may increase image noise and variability in quantifying MR-FDG and DV-FDG.

This study was prospective but conducted at a single center, with a relatively small cohort size. The requirement for patients to be scheduled in the first position in the morning, the need for increased staffing levels to supervise the lengthy scan taking place earlier than routine scanning and the limited number of patients reaching a tertiary care center without an established diagnosis contributed to a slow recruitment. Therefore, the results, especially the cut-off values, require validation in larger, multi-center cohorts. Additionally, these cut-off values should be applied exclusively to the PET/CT scanner and software applied in this study, as device-specific software and hardware variations in long axial field-of-view PET systems may significantly influence MR-FDG and DV-FDG measurements.

The study did not adjust for potential confounding factors, including biological (histological lesion type, BMI, residual variation in blood glucose) and technical (the assumption of irreversible tracer uptake, the non-invasive estimation of the input function, and partial volume effects) factors. These may have influenced FDG kinetics and should be addressed in multivariate analyses in future, larger studies.

The relatively small number of malignant lymph nodes and lung lesions led to broad steps in the AUC with limited coordinate points. Consequently, specificity calculations had to be performed on the 90.5% sensitivity threshold for lung lesions and the 92% threshold of lymph nodes rather than the usual 90% sensitivity.

## 5. Conclusions

Quantification of metabolic rate and distribution volume in total body Patlak PET differentiated between malignant and benign lung lesions and lymph nodes with high accuracy in this prospective, single-center trial. Multi-center validation will determine the generalizability of these findings. However, the choice of quantification method has a significant impact on the measurement of metabolic rate and distribution volume measurements. Therefore, tailoring cutoff values to specific quantification methods is essential for accurate clinical interpretation. In addition, standardized quantification methods are crucial for longitudinal and multicenter studies. For retrospective comparisons, the proposed conversion factor of 40% seems to be sufficient to compare the most commonly used “mean” and “max” quantification methods for MR-FDG and DV-FDG measurements in lung tumors.

## Figures and Tables

**Table 1:** Lymph node [^18^F]FDG activity.

Lymph Node (n=65)		Mean	Sd	Min	Max
SUV	mean	3.42	4.60	0.63	21.26
					
MR-FDG	mean	3.85	6.58	0.40	29.32
(µmol/(min × 100 mL))	max	5.40	9.60	0.57	43.11
	peak	3.92	6.37	0.63	32.79
					
DV-FDG	mean	57.02	35.97	12.95	172.63
(%)	max	78.18	63.76	19.35	309.93
	peak	64.08	37.90	16.70	193.10
					
CT Volume (mL)		2.05	6.40	0.03	50.72

**Table 2:** Lung lesion [^18^F]FDG activity.

Lung Lesion (n=33)		Mean	Sd	Min	Max
SUV	mean	6.32	5.52	0.93	23.69
					
MR-FDG	mean	10.54	12.53	0.73	57.81
(µmol/(min × 100 mL))	max	15.30	16.78	1.20	65.09
	peak	12.39	14.27	1.00	56.01
					
DV-FDG	mean	98.34	78.47	19.90	289.14
(%)	max	149.44	117.85	30.30	437.01
	peak	104.80	79.25	25.35	332.73
					
CT Volume (mL)		32.50	57.95	0.23	253.00

**Table 3:** Comparison of quantification methods mean, max and peak for the metabolic rate (MR) and distribution volume (DV) in lymph nodes. SD: Standard Deviation.

		Absolute				Percent			
		Mean Diff	SD	−1.96 SD	+1.96 SD	Mean Diff	SD	−1.96 SD	+1.96 SD
MR-FDG	Mean-Peak	−0.07	1.37	−2.77	2.62	−10.78	16.75	−43.61	22.05
(µmol/(min	Mean-Max	−1.55	3.13	−4.68	1.58	−26.40	13.79	−53.43	0.63
× 100 mL))	Max-Peak	1.47	3.48	−5.21	8.15	15.66	17.70	−19.03	50.35
									
DV-FDG	Mean-Peak	−7.06	9.13	−24.95	10.82	−14.11	16.30	−46.06	17.84
(%)	Mean-Max	−21.16	30.38	−80.71	38.39	−25.42	17.45	−59.62	8.78
	Max-Peak	14.09	27.14	−39.10	67.28	11.40	16.41	−20.76	43.56

**Table 4:** Comparison of quantification methods mean, max and peak for the metabolic rate (MR) and distribution volume (DV) in lung lesions. SD: Standard Deviation.

		Absolute				Percent			
		Mean Diff	SD	−1.96 SD	+1.96 SD	Mean Diff	SD	−1.96 SD	+1.96 SD
MR-FDG	Mean-Peak	−1.85	3.49	−8.70	4.99	−12.01	12.23	−35.98	11.96
(µmol/(min	Mean-Max	−4.76	5.04	−14.65	5.13	−40.87	5.69	−52.02	−29.72
× 100 mL))	Max-Peak	2.91	3.31	−3.58	9.39	29.10	12.20	5.19	53.01
									
DV-FDG	Mean-Peak	−10.64	17.24	−44.44	23.15	−14.15	15.93	−45.37	17.07
(%)	Mean-Max	−56.74	51.83	−158.34	44.85	−39.26	7.68	−54.31	−24.21
	Max-Peak	46.10	47.21	−46.43	138.62	25.29	14.58	−3.29	53.87

**Table 5:** Diagnostic performance of quantification methods mean, max and peak in lymph nodes (n=65).

		AUC	Std. Error	95% CI	*p*-Value
MR-FDG	mean	0.987	0.011	0.966–1.000	<0.01
(µmol/(min × 100 mL))	max	0.994	0.007	0.980–1.000	<0.01
	peak	0.991	0.009	0.974–1.000	<0.01
					
DV-FDG	mean	0.948	0.028	0.893–1.000	<0.01
(%)	max	0.975	0.016	0.943–1.000	<0.01
	peak	0.972	0.018	0.938–1.000	<0.01

**Table 6:** Diagnostic performance of quantification methods mean, max and peak in lung lesions (n=33).

		AUC	Std. Error	95% CI	*p*-Value
MR-FDG	mean	0.893	0.055	0.785–1.000	<0.01
(µmol/(min × 100 mL))	max	0.893	0.056	0.784–1.000	<0.01
	peak	0.905	0.051	0.805–1.000	<0.01
					
DV-FDG	mean	0.813	0.074	0.668–0.959	<0.01
(%)	max	0.812	0.074	0.666–0.957	<0.01
	peak	0.825	0.072	0.684–0.966	<0.01

## Data Availability

The data that support the findings of this study are available from the authors, but restrictions apply to the availability of these data, which were used with ethical approval restricted to this study. Data are available from the authors upon reasonable request and with prior ethical approval and under a data transfer agreement.

## Abbreviations

The following abbreviations are used in this manuscript:

AUC	Area under the curve
CONSORT	Consolidated Standards for Reporting Trials
CT	Computed tomography
DV	Distribution volume
FDG	Fludeoxyglucose
PET	Positron emission tomography
MR	Metabolic rate
MRI	Magnetic resonance imaging
PSF	point-spread-function
ROC	Receiver operating characteristic
SUV	Standardized uptake value
TAC	Time-activity curve
TOF	Time-of-flight
VOI	Volume of interest
WB	Whole body

## Appendix B. Patient Details

**Table A1:** Details of study participants regarding gender, age, height, weight, and lung lesion biology. NSCLC: Non Small Cell Lung Cancer.

Patient No.	Gender	Age	Size in cm	Body Weight in kg	Lung Lesion Biology
1	w	54	171	59.6	Pneumonia
2	m	81	189	71.4	Chronic lymphocytic leukemia
3	w	56	170	70.0	Not further classified
4	m	75	168	90.7	Adenocarcinoma
5	m	61	187	133.3	Hematoma
6	m	58	175	75.9	Adenocarcinoma
7	m	64	172	77.7	Cryptogenic organizing pneumonia
8	m	78	177	70.8	Adenocarcinoma
9	w	50	173	79.1	Adenocarcinoma
10	m	82	180	68.2	Benign pulmonary nodule not further classified
11	m	66	188	115.7	Adenocarcinoma
12	m	79	175	71.4	Inflammatory myofibroblastic tumor
13	m	69	168	67.2	Small cell lung cancer
14	w	71	164	67.8	Adenocarcinoma
15	w	73	168	90.0	Adenocarcinoma
16	m	77	158	49.3	Not further classified
17	w	76	153	80.4	Carcinoid of the Lung (Ki67: 1.2%)
18	w	60	170	80.5	Pleural dysplasia
19	m	41	190	107.0	Hamartoma
20	m	69	180	81.0	Acinar adenocarcinoma
21	w	57	157	66.1	Acinar adenocarcinoma
22	w	56	155	72.7	Hamartoma
23	w	56	160	71.3	Sarcoidosis
24	w	73	165	57.7	No target lesion
25	w	57	158	68.7	NSCLC
26	m	52	180	57.0	Pneumonia
28	m	61	184	49.2	NSCLC
29	m	66	187	105.2	NSCLC
30	w	69	139	56.2	Sarcomatoid carcinoma
31	m	59	168	106.9	Acinar adenocarcinoma
32	w	54	157	62.1	Acinar adenocarcinoma
33	m	73	187	95.5	Adenocarcinoma
35	m	54	179	86.5	Necrotizing granuloma
36	m	65	185	80.1	NSCLC
